# Risk of genotoxic damage in schoolchildren exposed to organochloride pesticides

**DOI:** 10.1038/s41598-020-74620-w

**Published:** 2020-10-16

**Authors:** Gerardo Alfonso Anguiano-Vega, Linette Hazel Cazares-Ramirez, Jaime Rendon-Von Osten, Alma Patricia Santillan-Sidon, Celia Gloria Vazquez-Boucard

**Affiliations:** 1 Molecular Biomedicine Laboratory, Facultad de Ciencias Químicas, Universidad Juárez del Estado de Durango, Durango, Mexico; 2 Laboratory of Identification of Persistent Organic Pollutants, EPOMEX, Universidad Autónoma de Campeche, Campeche, Mexico; 3 Proteomic and Genetic Toxicology Laboratory, Centro de Investigaciones Biológicas del Noroeste, Calle IPN 195, Playa Palo de Santa Rita Sur, 23096 La Paz, BCS Mexico

**Keywords:** Cell biology, Environmental sciences, Risk factors

## Abstract

This study identified and determined organochloride pesticide (OCs) concentrations in hair samples from children at two elementary schools: one exposed to fumigations in agricultural fields, the other unexposed. Three concentrations of OCs levels in the hair were compared (high, medium, low), and total nuclear abnormalities in buccal cells were determined: micronuclei (MNi), condensed chromatin, karyorrhexis, pyknosis, binucleate cells, karyolysis, lobed nuclei, and apoptosis. No significant differences were found for the presence of MNi between the schoolchildren from the exposed and unexposed schools, but the prevalence of OCs in both schools was over 50%, as well as the frequencies of MNi in the children were over 58%. Findings show a significant difference between the frequency of MNi in the total sample of schoolchildren (exposed school + unexposed school) in relation to the concentration of OCs detected in their hair. The children from exposed school that showed the higher concentrations of OCs in hair had higher levels of genotoxic damage in the buccal cells; compared against children with lower concentrations of OCs. The most frequent nuclear abnormalities in the exposed children were lobed nuclei (79.4%), binucleate cells (66.66%), apoptosis (65.07), and MNi (58.7%). We determined the prevalence ratio (PR) and prevalence odds ratio (POR) for the presence of MNi in buccal cells in relation to the OCs concentrations in the hair samples. Both ratios were high for MNi [PR 3.93, 95% confidence interval (CI) 1.97–7.84, *p* = 0.0003; and POR 7.97, 95% CI 2.62–24.28, *p* = 0.0003], indicating a 7.97 times greater risk that the exposed children will present > 0.2% of MNi when OCs concentrations exceed 0.447 μg/g. These indicators may be useful biomarkers of genotoxic damage in children exposed to persistent, highly-toxic compounds. Results suggest the potential risk to which those schoolchildren are exposed on a daily basis due to fumigations in nearby agricultural fields.

## Introduction

Both civil society and the scientific community are deeply concerned about the effects of pesticides on human health since applications of these substances can negatively impact human—and other—populations living near cultivated fields^1^. Vulnerable localities are often affected intensely because exposure is continuous and indiscriminate, and because they are impotent in the face of the enormous economic interests involved, which are largely indifferent to the damage they cause^2^. This situation arises mainly because the fieldworkers who apply the substances have inadequate knowledge of their properties and correct handling procedures, compounded by the fact that the responsible authorities fail to control their sale and use, and tend to minimize their effects^3^.

Children constitute the most vulnerable sector affected by these contaminants because their corporal systems are still developing and undergoing rapid changes, and because their detoxification processes have not matured. Also, pesticides can be present in the environment in soils, air and water, so they can affect children through respiration, food ingestion, and contact with the skin, inside or outside the home. Children’s exposure and susceptibility to these substances is greater, as well, because they tend to spend more time outdoors where they may come into contact with polluted dust and ground. Finally, they consume more possibly contaminated food and drink per kilogram of body weight and breathe more air than adults, but lack the experience necessary to protect themselves, especially in the early years of life^4^.

Organochloride compounds (OCs) stand out among pesticides due to of their capacity for persistence, accumulation, and biomagnification in organisms. The effects of OCs, however, appear only after long periods of chronic exposure, so they are difficult to detect. The biotransformation of OCs is slow and causes cumulative effects in people exposed^5^. Studies conducted with infant populations have focused on biomonitoring OCs in various matrices (blood, fatty tissue, dust, urine, hair) and on the effects of exposure to pesticides in general^6–10^. Little information is available on the toxic effects of OCs in children, but an association with an increased prevalence of infant leukemia, endocrine alterations, neurological development, delays in cognitive capacity, and autism is recognized^9,11^.

The International Agency for Research on Cancer (IARC)^12^ classifies some pesticides as carcinogenic or possibly carcinogenic for humans because they can produce genotoxicity, endocrine and chromosomal alterations, as well as mutations and signaling anomalies in embryonic or somatic cells^13,14^. Epidemiological studies provide evidence of the association between pesticide exposure and pediatric leukemia^14,15^, which represents 30% of all childhood cancers^16^. The risk of brain cancer, Wilm’s tumors, Ewing’s sarcoma, and germinal cell tumors has also been related to pesticide exposure^13,15^.

Some OCs are considered endocrine disruptors because they can interfere with the reproductive system by blocking or neutralizing hormonal actions and producing genital abnormalities in children and adolescents (e.g. cryptorchidism, hypospadias). The impact of endocrine disruptors on health remains unknown, though a relation to diabetes mellitus, obesity, autoimmune diseases, and cancer has ben mentioned^17^.

In summary, scientific evidence exists to support the hypothesis that pesticide exposure in general at both the occupational (in countries where children work in the agricultural sector) and residential levels (homes, schools, etc.) can be a major risk factor for exposed children that: (1) contributes to the incidence of serious diseases; (2) affects the growth and development of the reproductive apparatus; and (3) causes neurological and metabolic alterations^5–9^, though the risk attributable to each one of these factors and its associated elements is not yet clear.

Cytogenic biomonitoring is considered an integral part of good medical surveillance in people exposed to pesticides because it allows evaluating the potential risk of exposure to environmental genotoxic compounds, the early detection of side effects and chronic degenerative diseases, and the prevention of cancer development^18^.

The presence of micronuclei (MNi) and other cellular abnormalities in epithelial cells are considered biomarkers of genotoxic damage and genomic instability, produced by DNA damage or changes and alterations in the number or structure of chromosomes. If the genotoxic damage is not repaired naturally through cellular repair mechanisms, or if the altered cell is not eliminated, there is a danger that the defective cell will continue to replicate, producing alterations in its metabolic or physiological functions. This type of damage is considered one of the main mechanisms of chronic diseases in contexts of carcinogenesis and teratogenesis^19^.

In view of the paucity of information on the effects of OCs on infant populations potentially exposed to such contaminants, our objective was to evaluate their exposure (via chromatographic analysis of hair) and the risk of genotoxic damage (presence of MNi and other nuclear abnormalities in buccal cells) in schoolchildren exposed to fumigations with chemical compounds in agricultural fields located in front of an exposed school. This biomonitoring study was conducted in response to the concerns of parents and school authorities, and because of the strong odors that, almost daily, signal that workers are applying fumigants that may be harmful to schoolchildren.

## Materials and methods

### Ethical considerations

After obtaining authorization from the Principals of both schools, the children’s parents signed an informed consent letter and, at the same time, answered a questionnaire designed to gather personal information and data on risk factors. For each donor we gathered information on: (a) demographics (age, place of residence); (b) medical history (diseases, family antecedents of cancer); (c) parents’ occupations and time of exposure to chemicals; (d) parental smokers; (e) lifestyle habits (diet); (f) X-ray exposure in the last six months; (g) origin of drinking water; and (h) consumption of soft drinks. This study is part of the Project entitled “Identification of biomarkers of genetic damage in human populations exposed to pesticides”. Because the research protocol requires only non-invasive sampling methods, it was authorized by the Bioethics Committee of the Universidad Nacional Autónoma de México (Mexico City) on 11 February 2014.

### Study population

Todos Santos is a small (7000 inhabitants), isolated (80 km from the nearest urban space), town near the Pacific Ocean on the Baja California peninsula. Devoted essentially to ecological tourism, it has no industrial activity, and is considered practically a pristine site. The only source of contamination identified to date is a sector of agricultural activity centered primarily on chili pepper cultivation. A group of 87 children, ranging in age from 6 to 13 years with a minimum residence in the region of 5 years and at least 3 years of schooling (kindergarten and elementary school) was recruited and an observational cross-sectional study was realized. The school group that was exposed to agrochemicals consisted of 63 children (from a school situated some 70 m from a strip of land covering around 15 hectares of conventional pepper cultivation), while the reference group (children from an unexposed school located in the center of town, around 2 km from the exposed school) was made up of 24 students. Since these families are not directly affected by fumigations, the parents of the children who attend the reference school showed less interest in participating in the study.

### Sampling procedure

A lock of hair weighing approximately 1.0 g and 3 cm in length was cut from each participant (exposed children and controls) as close as possible to the nape of the neck, to detect OCs using gas chromatography. The validity and importance of hair analyses as a reliable tool for use in epidemiological studies related to the adverse effects of pesticides on human health has been amply validated^20–23^. At the same time, buccal cells samples were taken from each child using a scraper with a wooden applicator. Those samples were subjected to genotoxic studies by micronuclei assay.

### Gas chromatography

After washing in 1% triton, the hair samples were rinsed in distilled water and left to dry at room temperature for 12 h before performing chromatographic analysis^21^. Negative control analyses were conducted with each 15-sample lot to determine major recovery percentages at 90%. For identification and quantification, we utilized standard certificates of a mixture of OCs (Supelco 47426-U CLP Organochlorine Pesticides Mix). Samples were reconstituted to 50 mL for later gas chromatographic analysis using an electron capture detector (GC-ECD).

Micronuclei assay: prior to taking the buccal scraping samples, all the children rinsed their mouths 3 times with purified water. At that point, a sterilized, cotton-tipped applicator with wooden handle was passed gently with circular, ascending movements over the inside of the cheeks. Once extracted, the cells were deposited on frosted slides treated with 30 μl of deionized water, and cell smears were made. The samples were left to dry under open air in an area protected from dust and solar light. The next step was to fix the cells by applying 30 μl of absolute ethanol. Once the ethanol had evaporated, the buccal cell samples were stored on plastic lamella holders for conservation. The samples were evaluated using a double-blind approach to eliminate observer tendency. Fluorescence microscopy was used to perform the cell count and classification of the abnormalities detected in 500 cells per slide, performed in duplicate (i.e., 1000 total cells per subject). Ethidium bromide staining (0.1 μg/μl) was applied to observe and quantify total abnormal cells (TAC) using a Leica DL 200 fluorescence microscope with a 515–560-nm excitation filter and a 590-nm emission filter at 200 × and 400 × magnification. Cell evaluation was performed following established criteria^24^.

### Statistical analyses

Data were analyzed using descriptive statistics and a Student’s T test to compare means between the two study groups, with a chi square test to compare between-group frequencies. A Mann–Whitney U test was applied to compare variables with non-parametric distribution, and a Kruskal Wallis test to compare tertiles. Linear regression and correlation analyses were conducted by calculating Pearson’s correlation coefficient (Pearson’s r) in order to recognize associations between OC concentrations and the frequencies of cellular abnormalities. All analyses were carried out using the SPSS statistical package, v.15. Estimates of prevalence (PR), and prevalence odds ratios (POR), were based on a 2 × 2 contingency table. Finally, a chi square test^25^ was performed with Epidat 3.1. In order to assess the frequency differences of cell abnormalities between groups of children with high and low concentrations of total OCs, a cut-off point (COP) was determined that corresponded to the average of total OCs in the total population (0.447 μg/g) (COP > 0.447 μg/g). Two 2 × 2 contingency tables were constructed. The cut-off points for each one of the cellular variables were the values of the average obtained from the population for TAC (COP > 1.4%) and MNi (COP > 0.2%). The contingency tables were evaluated by the chi-square test to determine significant differences (*p* > 0.05). In addition, the PR and POR values were calculated for each table.

## Results

No significant differences were found in the age (*p* = 0.895) or gender (*p* = 0.061) of the children in the two groups. Chromatographic analysis detected 19 OCs in the children’s hair: hexachlorocyclohexane isomers alpha (αHCH), beta (βHCH), delta (δHCH) and gamma (γHCH), endosulfan sulfate (ES), endosulfan IA (ES IA), endosulfan IIB (ES IIB), aldrin (AL), dieldrin (DIE), endrin (END), endrin ketone (ENK), endrin aldehyde (ENA), cis-chlordane (CC), trans-gamma chlordane (TG), heptachlor (HE), epoxyheptane (EH), dichlorodiphenyltrichloroethane (DDT), dichlorodiphenyldichloroethane (DDD), and dichlorodiphenyldichloroethylene (DDE) isomers.

**Figure 1:**
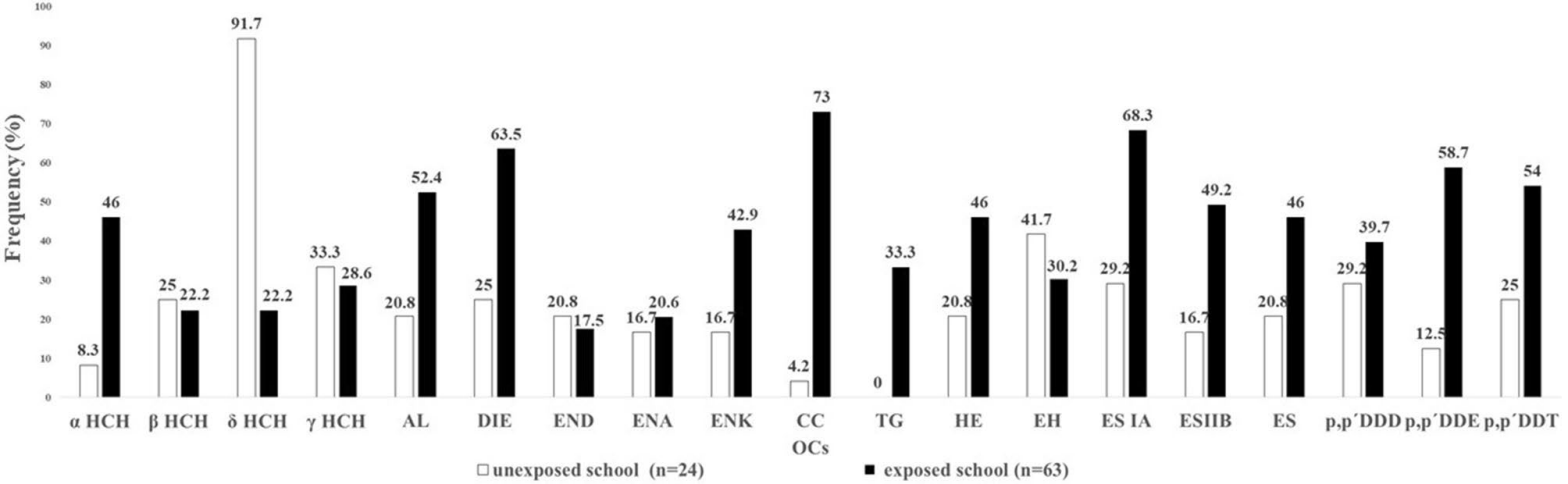
Frequency (%) of OCs in the hair of the study population from the exposed and unexposed schools. αHCH, hexachlorocyclohexane alpha; βHCH, beta; δHCH, delta; γHCH, gamma; AL, aldrin; DIE, dieldrin; END, endrin; ENA, endrin aldehyde; ENK, endrin ketone; CC, cis-chlordane; TG, trans-gamma chlordane; HE, heptachlor; EH, epoxyheptane; ES IA, endosulfan IA; ES IIB, endosulfan IIB; ES, endosulfan sulfate; DDD, dichloro diphenyl dichloroethane; DDE, dichloro diphenyl dichloroethylene; DDT, dichloro diphenyl trichloroethane.

**Figure 2:**
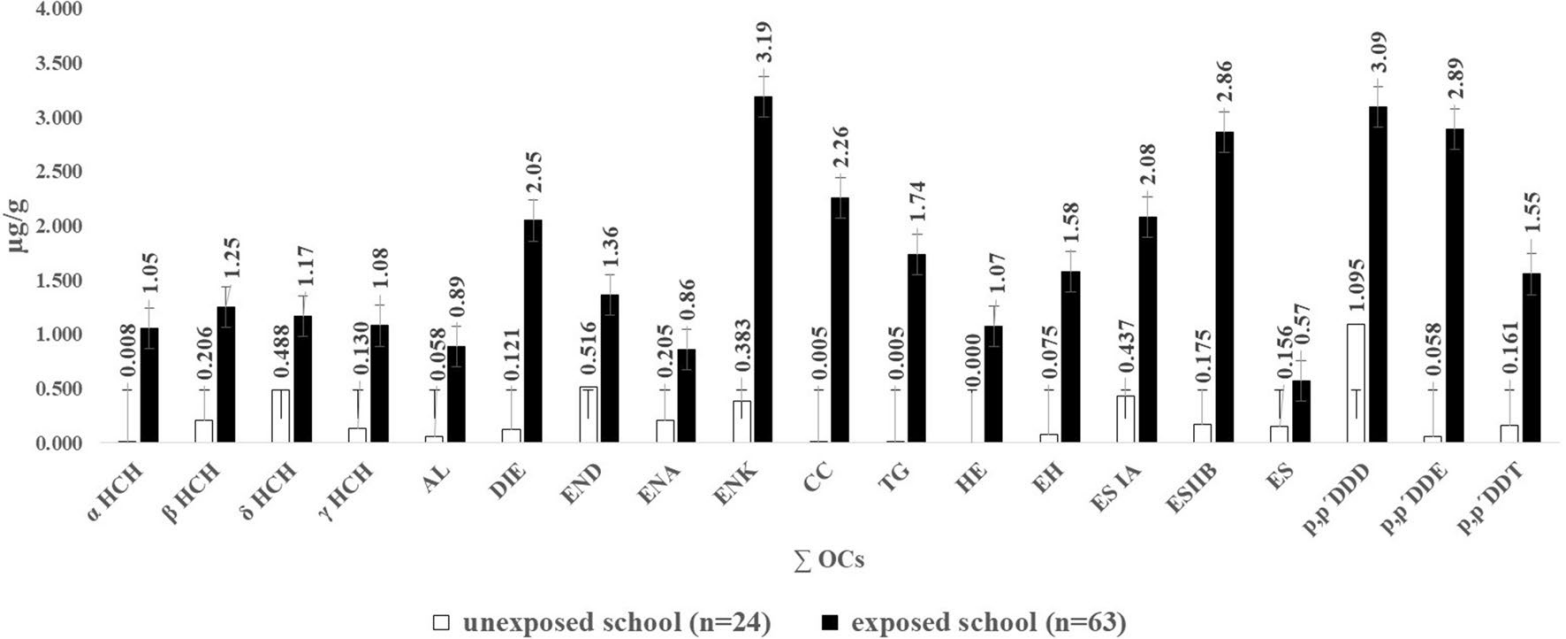
Concentration (μg/g) of total sum OCs in the hair of the study population from the exposed and unexposed schools. αHCH, hexachlorocyclohexane alpha; βHCH, beta; δHCH, delta; γHCH, gamma; AL, aldrin; DIE, dieldrin; END, endrin; ENA); ENA, endrin aldehyde; ENK, endrin ketone; CC, cis-chlordane; TG, trans-gamma chlordane; HE, heptachlor; EH, epoxyheptane; ES IA, endosulfan IA; ES IIB, endosulfan IIB; ES, endosulfan sulfate; DDD, dichloro diphenyl dichloroethane; DDE, dichloro diphenyl dichloroethylene; DDT, dichloro diphenyl trichloroethane.

The OCs that appeared at frequencies greater than 50% in the exposed group were CC (73%), ES IA (68.3%), DIE (63.5%), DDE (58.7%), DDT (54%), and AL (52.4%), while in the control group only δHCH (91.7%) was detected at this level (Fig. 1). The most significant concentrations identified in the exposed group were ENK (3.19 µg/g), DDD (3.09 µg/g), DDE (2.89 µg/g), and ES IIB (2.86 µg/g), while in the control group they were DDD (1.09 µg/g), δHCH (0.48 µg/g), ES IA (0.43 µg/g), and ENK (0.38 µg/g) (Fig. 2).

**Figure 3:**
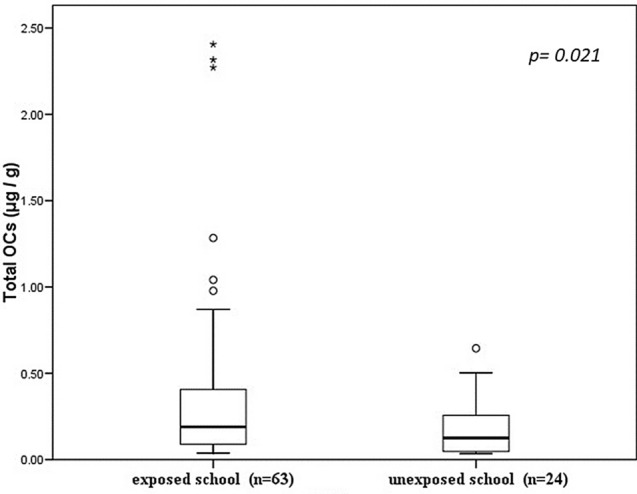
Concentration of total OCs (μg/g) in the two study groups. Values are expressed as medians (inter-quartile range); a Mann–Whitney U test exposed school versus unexposed school.

The total and mean OC concentrations detected in the exposed schoolchildren were 28.19 and 0.9447 µg/g, respectively. These values were statistically significant when compared to the results obtained with the unexposed controls: 4.39 µg/g and 0.182 µg/g (*p* = 0.021) (Fig. 3).

**Figure 4:**
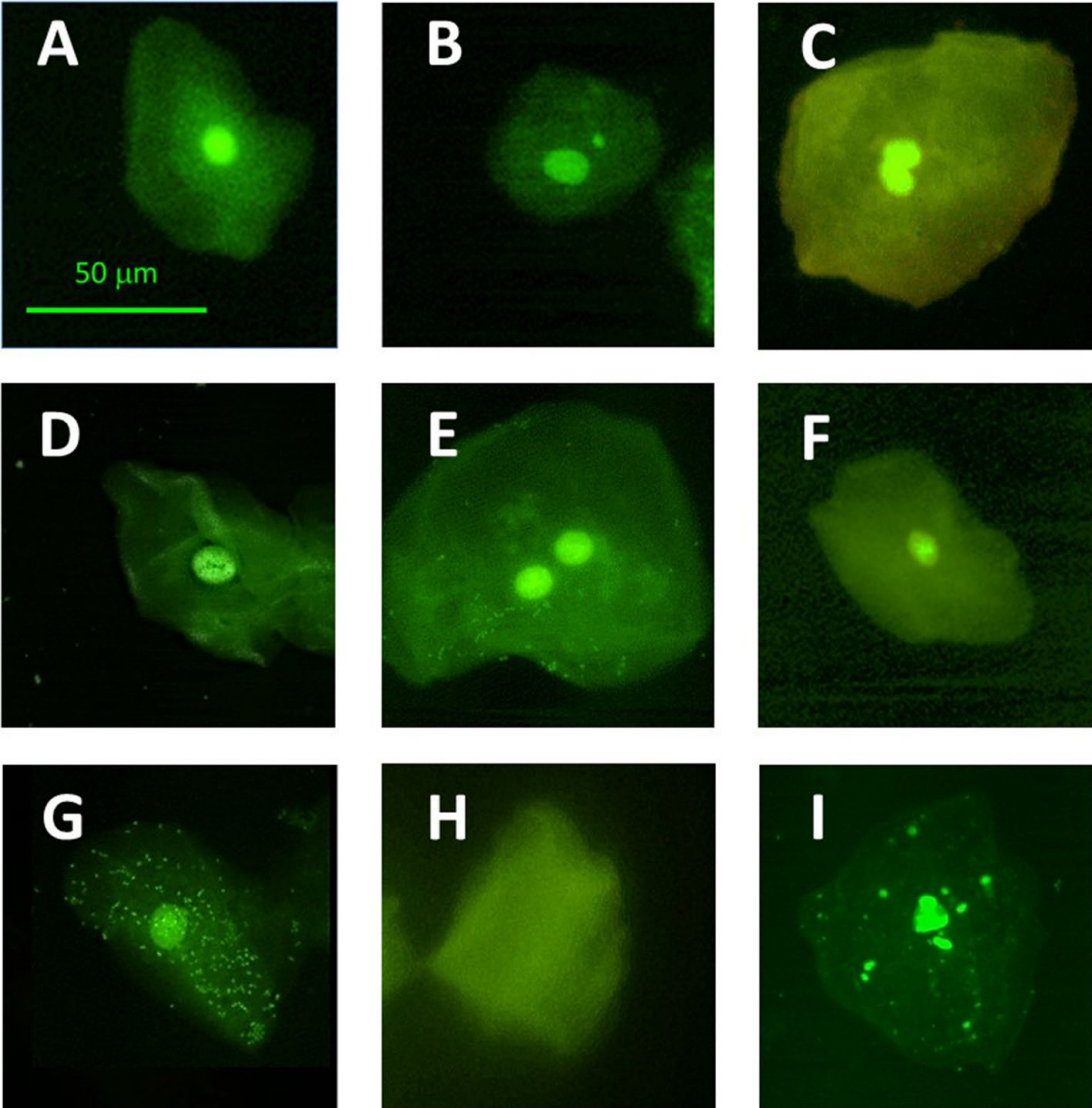
Microscope images of the mayor nuclear abnormalities observed in the micronuclei assay. (**A**) Normal cell, (**B**) Micronuclei, (**C**) Lobed nuclei, (**D**) Condensed chromatin, (**E**) Binucleated Cell, (**F**) Pyknosis, (**G**) Karyorrhexis, (**H**) Karyolysis, (**I**) Apoptosis.

**Figure 5:**
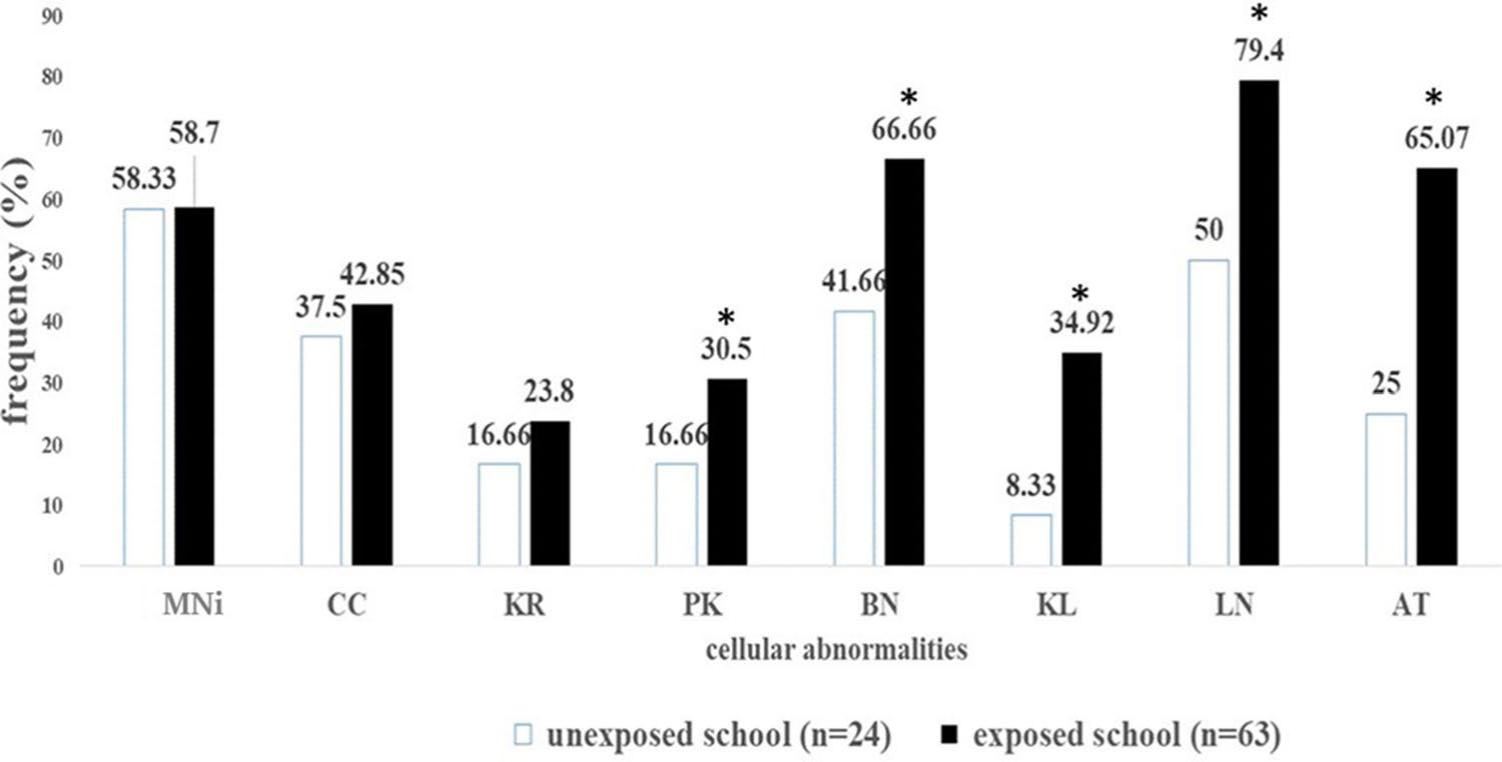
Frequency (%) of nuclear abnormalities in unexposed and exposed schoolchildren: Micronuclei (MNi), Condensed chromatin (CC), Karyorrhexis (KR), Pyknosis (PK), Binucleated cells (BN), Karyolysis (KL), Lobed nuclei (LN), and Apoptosis (AT). *Significant differences (*p* < 0.05) by chi square test.

The genotoxic study carried out with the buccal cells of the exposed and unexposed children identified the following total abnormal cells (TAC): micronuclei (MNi), condensed chromatin (CC), karyorrhexis (KR), pyknosis (PK), binucleate cells (BN), karyolysis (KL), lobed nuclei (LN), and apoptosis (AT) (Fig. 4). The most prevalent nuclear abnormalities in the exposed population were LN (79.4%), BN (66.66%), AT (65.07%), and MNi (58.7%), while for the unexposed children they were MNi (58.33%), LN (50%), BN (41.66%), and CC (35.7%). The frequencies of nuclear abnormalities that showed significant differences between the unexposed and exposed schools were PK, BN, KL, LN, and AT (Fig. 5).

**Figure 6:**
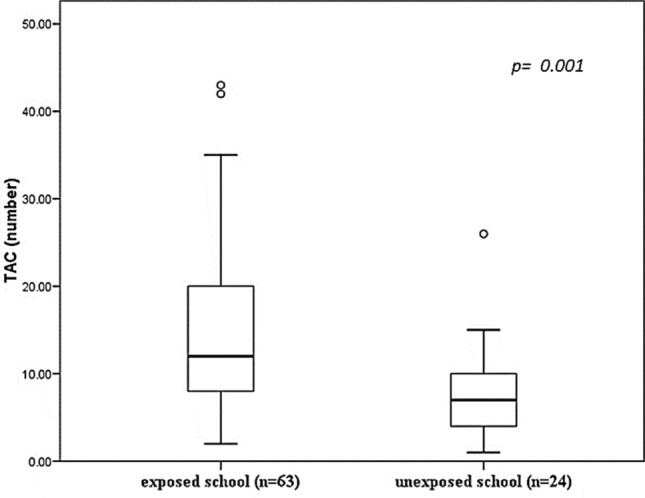
Number of total abnormal cells (TAC) in the two study groups. Values are expressed as means ± standard deviation; student’s T test between exposed school versus unexposed school.

When comparing the TAC frequencies between the groups of children from the exposed and unexposed school, they showed higher values for the exposed group (14.841) over the control (5.542) (*p* = 0.001) (Fig. 6).

The average number of nuclear abnormalities between the two study groups showed significant differences for BN, KL, LN, and AT. The nuclear abnormalities LN (5.190), BN (2.492), and AT (2.175) had the highest averages for the group of children from the exposed school (Table 1).

**Table 1:** Means and standard deviations of abnormal cells in children from exposed and unexposed schools.

	Unexposed school Mean ± SD	Exposed school Mean ± SD	*P*^a^
Micronuclei (MN)	1.167 ± 2.036	2.032 ± 3.600	0.166
Condensed chromatin (CC)	0.522 ± 1.123	0.873 ± 1.273	0.182
Karyorrhexis (KR)	0.292 ± 0.858	0.397 ± 0.890	0.615
Pyknosis (PK)	0.625 ± 1.789	0.683 ± 1.673	0.892
Binucleate cells (BN)	0.875 ± 1.393	2.492 ± 2.988	< 0.001
Karyolysis (KL)	0.083 ± 0.282	1.016 ± 2.004	< 0.001
Lobed nuclei (LN)	1.458 ± 2.734	5.190 ± 4.703	< 0.001
Apoptosis (AT)	0.541 ± 1.285	2.175 ± 2.791	< 0.001
Total abnormal cells (TAC)	5.542 ± 3.833	14.841 ± 9.479	< 0.001

^a^t student test of equality of means.

**Figure 7:**
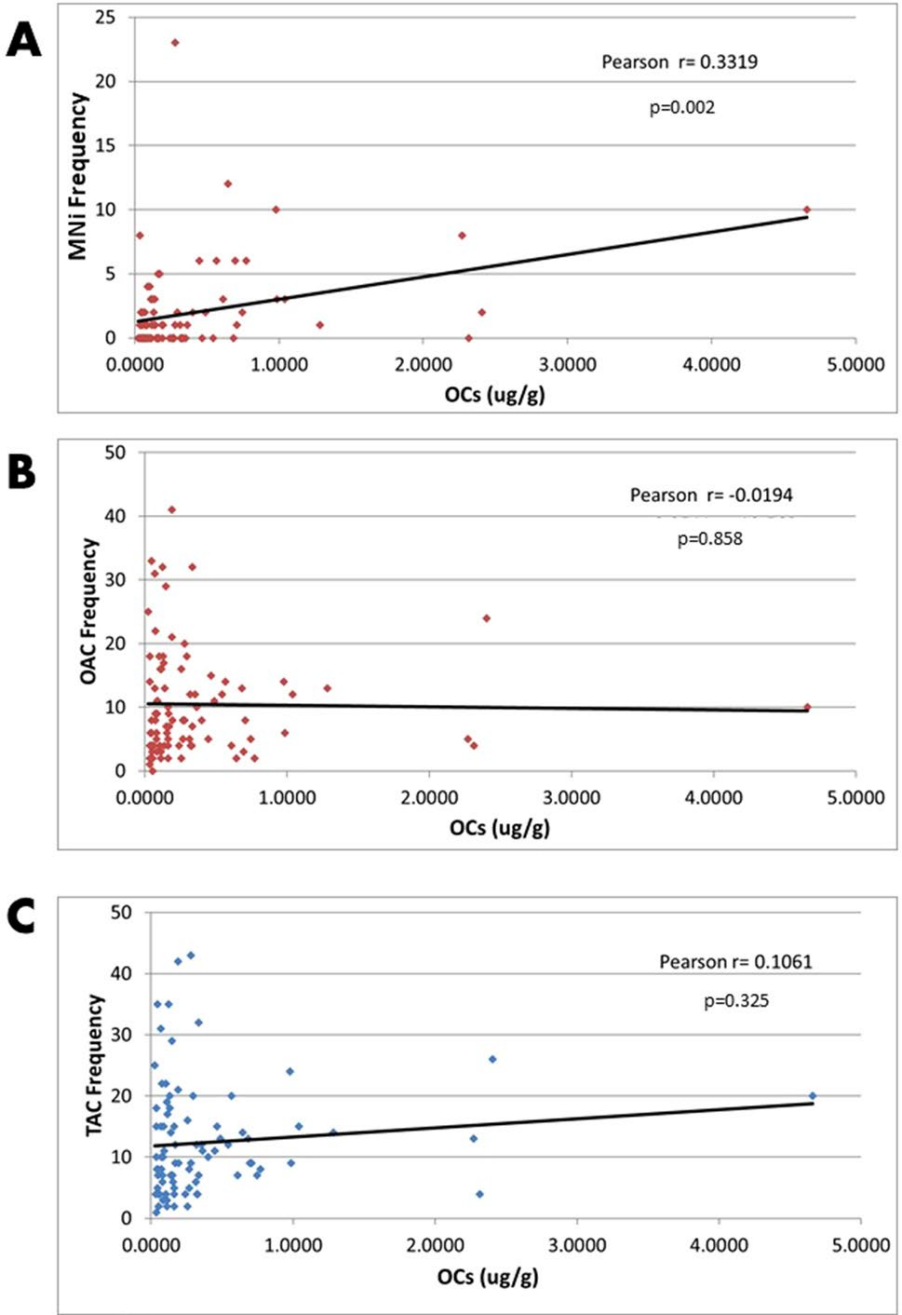
Correlation between abnormal cells versus total concentration of OCs in both study groups. (**A**) Micronuclei versus OCs, (**B**) other abnormal cells versus OCs; (**C**) total abnormal cells versus OCs.

In an attempt to identify the possible association between OC levels and the frequency of nuclear abnormalities, a logistic regression analysis was conducted and Pearson’s correlation coefficient (r) was calculated for MNi, TAC, and other abnormal cells (OAC) versus total OC concentrations. Only the correlation for MNi versus OCs showed statistical significance (*p* = 0.002) with a positive association r = 0.3319; TAC versus OCs and OCA versus OCs did not present statistical significance (*p* = 0.325, r = 0.1061) and (*p* = 0.858, r = 0.0019) respectively (Fig. 7).

**Figure 8:**
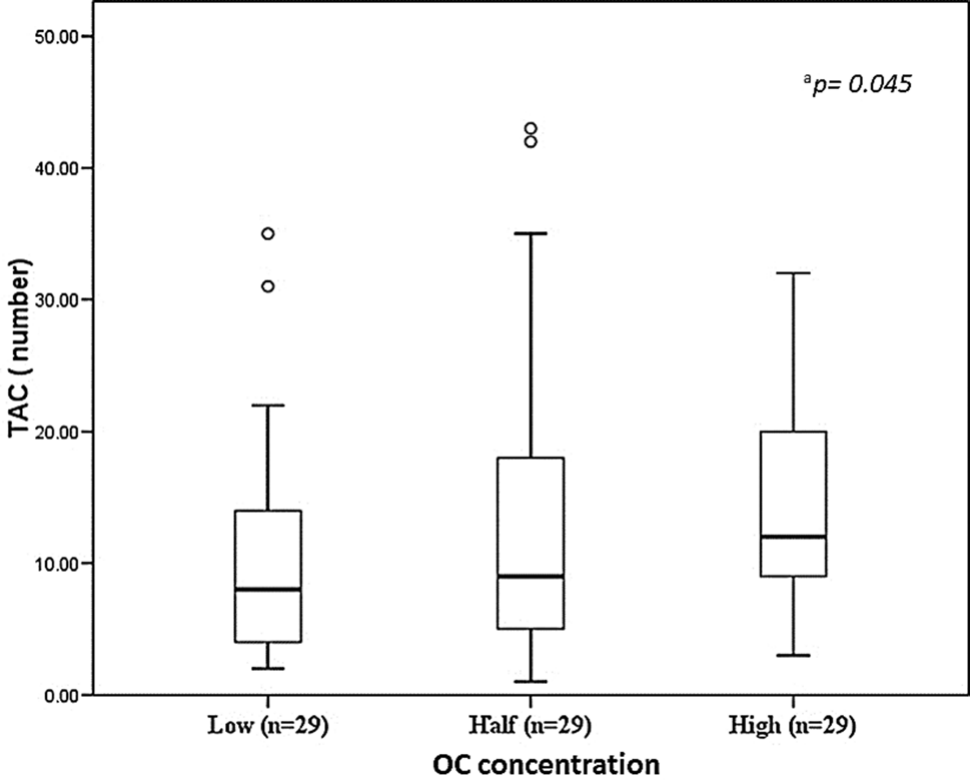
Number of total abnormal cells (TAC) in the study population stratified by OC concentration tertiles. Values are expressed as medians (inter-quartile range); Mann–Whitney U test; ^a^low versus high concentration group (*p* = 0.045).

The results for all participants (exposed + unexposed) were organized by tertiles, each with 29 individuals, as a function of total OC concentrations (tertile 1: concentrations of 0.0350–0.1006 µg/g; tertile 2: 0.1111–0.2807 µg/g; tertile 3: 0.2953–4.6593 µg/g) (*p* = 0.001), in order to obtain more ample, statistically-valid information on the genotoxic effects of these contaminants in relation to different levels of OC concentration. When the TAC and total OC concentrations were evaluated in the subjects organized by tertiles, observations showed higher numbers of TAC as OC concentrations increased, and that this was statistically-significant (*p* = 0.045) between the low- and high-concentration groups (Fig. 8).

The contingency table for MNi versus. total OCs showed a significant difference on the chi-square test (*p* = 0.0003) with high risk values of PR = 3.93, 95% confidence interval (CI) 1.97–7.84 and POR = 7.97, 95% CI 2.62–24.28. The contingency table for TAC versus total OCs showed no significant difference (*p* = 0.8240) on the chi-square test or significant PR and POR risk values: PR = 1.02, 95% IC 0.48–2.160, and POR = 1.03, 95% CI 0.35–3.01 (Table 2).

**Table 2:** Contingency tables for analysis of frequencies by total OCs, micronuclei and total abnormal cells; and calculate of relative risks and odds ratio in schoolchildren exposed to pesticides.

		Total OCs COP > 0.477 μg/g				
			Low	High	Total	*P*
Total OCs versus micronuclei	MNi COP > 0.2%	Low	58	8	66	
		High	10	11	21	
		Total	68	19	87	
	n = 87				χ^2^	0.0003
PR = 3.93 IC 95% (1.97–7.84) POR = 7.97 IC 95% (2.62–24.28)						

		Total OCs COP > 0.477 μg/g				
			Low	High	Total	*P*
Total OCs versus total abnor cell	TAC COP > 1.4%	Low	47	13	60	
		High	21	6	27	
		Total	68	19	87	
	n = 87				χ^2^	0.8240
PR = 1.02 CI 95% (0.48–2.16) POR = 1.03 CI 95% (0.35–3.01)						

OCs, organochlorines pesticides; COP, cut-off point; MNi, micronucle; ,χ^2^, chi-square test; PR, prevalence ratio; POR, prevalence odds ratio; CI, confidence interval.

### Ethics approval

This study was performed in full compliance with the principles of the Helsinki Declaration. It is part of the project entitled “Identification of biomarkers of genetic damage in human populations exposed to pesticides”. Because the research protocol required only non-invasive sampling methods, it was authorized by the Bioethics Committee of the Universidad Nacional Autónoma de México (Mexico City) on 11 February 2014. After obtaining authorization from the Principals of both schools, the children’s parents signed an informed consent letter and, at the same time, answered a questionnaire designed to gather personal information and data on risk factors.

## Discussion

### Presence of OCs in children

OCs are considered persistent organic compounds due to their high accumulation capacity, persistence, and biomagnification in organisms^5^. The monitoring procedure conducted found a significant increase of OCs in the hair of the children attending the school located close to the agricultural fields. Most of those OCs are either prohibited (AL, DIE, ENA, ENK, DDT, DDD, DDE) or restricted (αHCH, βHCH, γHCH, δHCH, ES IA, ES IIB) by both Mexican legislation^26–28^ and the Stockholm Convention^29^, of which Mexico is a signee. The IARC^12^ has classified some of the OCs identified in this study as: carcinogens (type 1) γHCH; or probable carcinogens (type 2A) DDT, DDE, DDD, aldrin, and dieldrin. It is important to note, as well, that other compounds identified—e.g. CC, HE, EH—are also considered harmful for human health, though they have not been prohibited or restricted.

It might be alleged that the contaminants detected could be attributable to previous exposures that led to accumulations in the children’s organisms. However, the 3-cm long hair samples can only reveal the OC concentrations to which the children were exposed during the 3-month period prior to sampling (since hair grows approximately 1 cm/month). Because of certain characteristics, hair provides information that cannot be obtained from other biological samples (e.g. blood or urine) due, primarily, to the prolonged exposure time detected (around 3 months), and the possibility of establishing a chronological profile. Urine and blood plasma only provide information related to recent exposures because persistent contaminants—like the OCs analyzed—are stored quickly in the fatty tissue of exposed organisms^30^.

Therefore, this study provides evidence that restricted and/or prohibited agrochemicals continue to be commercialized and applied on crops in Todos Santos, Baja California Sur, Mexico, although both state and federal agencies are entrusted with controlling their availability and use. This problematic affects numerous countries, because these compounds are so effective in protecting crops, but this simply reflects the reality that economic interests are placed above concerns for human health.

Another factor of concern is the high concentration of lindane (γ HCH) identified in the children from the control school, a substance that reflects the fact that families apply products to children’s hair to eliminate ectoparasites like lice and nits. Lindane is, in fact, an organochloride classified as carcinogenic for humans. Though our results reveal that 94% of the control children showed signs of exposure to HCH, the concentrations present in their hair samples were much lower than those registered for this compound in the exposed children.

No database is available that would allow us to verify the maximum allowable limits for OCs in humans, but as a reference we consulted the maximum allowable limits for residues established by the European Community for foods, animal foods, and foods of vegetable origin^31^. That document clearly indicates that the concentrations found in the present study exceed the allowable limits for residues (0.01 mg/kg) determined by default for all pesticides, though specific studies have not yet been performed for each one. In the event that the concentration of each OCs was found below the limits that this legislation considers “safe”, it is necessary to consider the health implications of chronic exposure to mixtures of these chemical compounds. even though that at the individual level each pesticide is below the corresponding limit, when mixed with other chemicals, they can present antagonisms to each other, which could enhance their effects^32^.

### Effects on children’s health

Cancer is a major cause of death in children worldwide, and the incidences recorded tend to increase over time, with leukemia being the most common form^33^. Several studies have shown that incidences of this disease increase in relation to pesticide exposure^34–39^. Other positive relations between pesticide exposure and cancer in children, though less statistically-significant, have been found in the incidence of retinoblastomas and bone cancer^40^, Wilm’s tumor^41^, kidney cancer^42^, and testicular cancer^43^. Also, the same types of cancer associated with exposure to agrochemicals in adults have been identified in children^32^. Regarding acute lymphoblastic leukemia, the general mechanisms that underlie induction of this disease are similar in adults and children, and include mutagenic processes and altered transcription factors, as well as hyperdiploidy^14^. Some studies have confirmed the association of OC concentrations in adults and children with the development of leukemia^44–46^.

The OCs: ES, DDT, DDE DDD are considered endocrine disrupters9 that are capable of causing adverse effects in the health of persons exposed, or their descendants, such as cancer during adulthood, cellular, structural, and functional anomalies (liver detoxification mechanisms, thyroid function, male sexual function) alterations in growth, and pubertal and psychomotor development^47–49^.

Exposure to DDE and ES has been associated with immunological alterations and the development of otitis and respiratory symptoms (obstructive and restrictive pulmonary diseases like asthma)^50–52^, and with the risk of contracting neurological disorders like autism and attention-deficit syndrome in children aged 3–10 years^53^. The existence of a relationship between levels of exposure to DDT and the frequency of breast cancer in women before age 50, when exposure occurred before age 14 has also been verified^54^. On the other hand, genetic polymorphisms in certain enzymes involved in the detoxification of CYPIA1, CYP2E1 and NQO1 can cause potentially modifiable effects that can increase the risk of leukemia^55,56^.

### Genotoxic effects

In contrast to our expectations, the results obtained in this study did not reveal a significant increase in the frequency of MNi in the group of exposed schoolchildren. Given the significant increase in the OC concentrations confirmed in the children from the exposed school, we expected to find a higher frequency of MNi than in the unexposed controls. Similar studies of the effects of pesticides on the frequency of MNi in buccal cells provide only inconsistent results^57^. Significant differences in the frequency of MNi have been demonstrated, for example, in women agricultural laborers with respect to an unexposed group^58^, while workers on banana plantations showed an increase in the frequency of micronuclei when compared to unexposed controls^59^. A study carried out with greenhouse workers found significant differences in nuclear alterations (MNi, BN,PK, KR) compared to a group that was not exposed to pesticides^60^; however, those studies did not conduct analyses of pesticide concentrations that might reveal a direct association, but only considered potential exposures based on a list of the pesticides utilized.

The correlation studies between total OC concentrations and the frequency of nuclear abnormalities herein showed that only the association for MNi had statistical significance, but none of the OAC and TAC which did not present statistical significance. These correlations demonstrate that MNi are the abnormalities that best represent exposure to pesticides, though observations showed that the frequencies of MNi were not very high, even at elevated concentrations of OCs, possibly due to the actions of repair processes or elimination of damaged cells. We also observed that certain children were more sensitive to exposure to OCs, as they presented high frequencies of MNi at low or moderate levels of pesticide concentrations. These conditions could explain why the Pearson’s r value did not approach 1.0. Other nuclear abnormalities also failed to show significant correlations, as their r values were close to zero. Finally, some studies have demonstrated moderate-to-low correlations between the frequency of MN and factors of genetic instability such as ionizing radiations, benzene, or tobacco smoke^61–63^.

The frequencies of TAC found in the exfoliated cells from epithelial buccal tissue may be the result of an alteration of the normal processes of cellular replication, which could be influenced by various factors in addition to exposure to OCs, as well as by the rhythm of cellular renewal. It is well-known that MNi form in the cells of the basal layer of epithelial tissue where cell division takes place, before migrating to the surface over the ensuing 5–14 days^18^. This process is not cumulative because the proliferative dynamics of the oral epithelium impede it. For this reason, it is not easy to relate nuclear alterations directly to the OC concentrations to which individuals are exposed. Nuclear alterations are a reflection of multiple processes and intra- and extracellular factors that convert them into a complex system. These variations depend, as well, on alimentation, gender, and age^64^.

Dividing the entire sample of participants in the study (exposed and unexposed children) into sub-groups (low, medium, and high OC concentrations), however, revealed a statistically-significant increase in TAC in the individuals with higher OC concentrations in their hair. The prevalences of MNi identified in both schools are important (58%), and the average value of the frequency of MNi in each child was 0.2%, close to the value that is considered the normal average of frequencies of micronuclei in children, which ranges from 0.1 to 3.6%^58,65^.

Other nuclear anomalies were observed during our TAC analysis, though they may occur during normal processes of cell differentiation, or be indicators of DNA damage, cytotoxicity, and cell death, when observed at high frequencies^18,66^. These abnormalities can be distinguished from normal cells either by alterations in the cytoplasm or the morphology of the nucleus. These alterations may include condensed chromatin (CC), karyorrhexis (KR), and karyolysis (KL), which are considered bioindicators of cytotoxicity; pyknotic (PK) or lobulated nuclei (LN) that indicate DNA damage; and binucleate cells (BN), which are indicators of cytocinesis^67–69^. The mechanism of the formation or biological significance of each one of the TAC have not been clarified, though studies have found high frequencies of TAC under pathological conditions (systemic lupus erythematosus, obesity, rheumatoid arthritis, various types of cancer, and hematological problems like immunoblastic lymphoma, Hodgkin’s disease, acute megaloblastic leukemia, chronic granulocytic, acute lymphocytic, lymphocytic anemia, multiple myeloma, and thrombocytopenic anemia, among others)^18^. These abnormalities are also observed in aging processes where the frequencies of MNi, NL, and BN are all elevated^21^.

The significant increase in TAC demonstrated in this study in the buccal cells of the children from the exposed school compared to those from the unexposed school, together with the increase in TAC in the group of high concentration versus the low concentration group, indicate the potential risk to which these schoolchildren are exposed due to daily fumigations in nearby agricultural fields. In effect, the prevalence ratio (PR = 3.93) and prevalence odds ratio (POR = 7.97) values determined between the OCs concentrations detected in the children’s hair and genotoxic damage indicate that OCs concentrations above 0.477 μg/g increase 7.97 times the risk of developing MNi greater than 0.2%. The comparison of groups exposed and unexposed to OCs by means of POR calculations is a statistical measure of basic risk assessment that makes it possible to establish the magnitude of the frequency of MNi according to the OCs concentrations detected in children's hair. The POR values calculated in our study are considered preliminary due to the small number of participants. Our search for bibliographical data that would allow corroboration was unsuccessful, so new studies are needed to corroborate the POR we determined and verify its use as a biomarker of exposure to high OCs concentrations in populations exposed through residence and/or occupation.

We can affirm, therefore, that the formation of MNi was sensitive to the greater presence of OCs, regardless of whether the children were from the exposed or the unexposed, control, school. Though some pesticides may not be genotoxic, they can interfere with signal transduction, resulting in hypermutability, genomic instability, the loss of control of cellular proliferation, and resistance to apoptosis, all of which cause neoplastic damage through their action on genetic material. It is these different modes of action that make it difficult to characterize these compounds as carcinogens. Genomic instability is a risk factor for all types of cancer, and the presence of MNi is considered a biomarker of this disease, so the risk to which the children studied herein are exposed is certainly a cause for concern, and reveals that it is urgent to distance them from the sources of emissions of agrochemicals.

## Conclusion

The present study allowed us to document the utilization of harmful OCs in the study region that constitute a high risk for human and environmental health. Some of these substances are prohibited and/or restricted internationally due to their demonstrated participation in the development of serious diseases. Likewise, we show that the children from the school close to the agricultural fields are affected by fumigations, as we observed OCs concentrations that were more statistically-significant than those recorded in children from the unexposed school. We observed more important genotoxic damage (MNi) in the individuals with higher concentrations of OCs in their hair. This finding allowed us to obtain a statistical measure of the basic evaluation of the risks to which these schoolchildren are exposed. Therefore, we can affirm that the health of the children who attend the school located near the cultivated fields is at a high risk for health problems today and in the future.

Among the greatest challenges in identifying the effects of agrochemicals is the fact that they are often very subtle, can be triggered at low concentrations, and may not become apparent until several years after exposure or even, in some cases, only in subsequent generations. In most cases, the concentrations found in our study fall within the limits that existing legislation considers “safe”, even though the health impacts of daily exposure to mixtures of chemical compounds with shared action mechanisms, even in small doses, are largely unknown. This means that diagnoses, prevention, and the rigorous determination of the potential effects of OCs are all difficult to obtain. On the other hand, monitoring of populations exposed to pesticides is not easy to carry out, due to the problems of conflicts and interests that do not allow obtaining an adequate number of participants. This is the reason why the present study should be considered preliminary and be repeated in more numerous exposed populations. Obviously, this increases the difficulty of evaluating their consequences for public health. Nonetheless, the principle of precaution—that is, implementing preventive actions in the face of reasonable scientific uncertainty in relation to environmental exposures that threaten human health—underscores the need to implement the following measures: (a) programming and conducting systematic biomonitoring in exposed populations; and (b) eliminating sources of contamination in residential zones near agricultural fields. Finally, the use and control of products that are harmful for human and environmental health must be strictly regulated and monitored by the agencies responsible.
